# Neurogenin 3 is important but not essential for pancreatic islet development in humans

**DOI:** 10.1007/s00125-014-3349-y

**Published:** 2014-08-14

**Authors:** Oscar Rubio-Cabezas, Ethel Codner, Sarah E. Flanagan, José L. Gómez, Sian Ellard, Andrew T. Hattersley

**Affiliations:** 1 Department of Pediatric Endocrinology, Hospital Infantil Universitario Niño Jesús, Instituto de Investigación Sanitaria La Princesa, Avda. Menéndez Pelayo 65, 28007 Madrid, Spain; 2 Institute of Maternal and Child Research, University of Chile, Santiago, Chile; 3 Institute for Biomedical and Clinical Science, University of Exeter Medical School, Exeter, UK; 4 Department of Pediatrics, Complejo Hospitalario Torrecárdenas, Almería, Spain

**Keywords:** Diabetes, Enteric anendocrinosis, Islet development, Neonatal diabetes, Neurogenin 3

*To the Editor:* Based on the findings that *Neurog3*-null mice fail to develop pancreatic islets [1] and lack enteroendocrine cells [2], this basic helix-loop-helix (bHLH) transcription factor is considered to be essential for differentiation of endocrine cells both in the pancreas and in the gut [3]. Limited data from human fetuses suggest that transient expression of *NEUROG3* in pancreatic progenitor cells from approximately 7 to 8 weeks post conception is followed by beta cell differentiation and immature islet formation by week 12 of fetal development [4, 5].

Homozygous loss-of-function mutations in *NEUROG3* were first identified in patients with a rare form of congenital malabsorptive diarrhoea that was due to enteric anendocrinosis [6]. Unexpectedly, the patients did not present with neonatal diabetes, suggesting that pancreatic islet development was at least partially spared, although they developed diabetes during late childhood. The authors hypothesised that an unidentified factor might compensate for the absence of functional neurogenin 3 (NEUROG3) and result in the production of some functional beta cells. Further studies showed that the reported mutations were hypomorphic rather than null mutations. Therefore, differences in *NEUROG3* gene dosage requirements could explain the observed differential effect on the intestinal and pancreatic phenotypes [7].

We recently identified biallelic null *NEUROG3* mutations in a patient with enteric anendocrinosis and permanent neonatal diabetes [8]. Although pancreatic tissue was not available for direct analysis, the presence of detectable blood C-peptide indicated that there was some endogenous insulin production at 4.5 years of age. Similar findings were later reported in a different patient [9]. Consequently, we hypothesised that, despite the pivotal role of *Neurog3* in pancreatic endocrine development in mice, complete NEUROG3 deficiency does not necessarily imply an absolute insulin deficiency in humans and, therefore, affected patients may present with diabetes at any age.

To test this hypothesis, the single coding exon of *NEUROG3* was amplified and sequenced from genomic DNA as previously described [8] in a further three probands with congenital malabsorptive diarrhoea and diabetes, regardless of the age at diagnosis of the latter. This study was conducted in accordance with the Declaration of Helsinki and approved by the local ethics committee. Written informed consent was obtained from the parents/guardians of the patients. Previously described homozygous missense mutations were identified in the three cases and a non-diabetic sibling of proband 3. Clinically unaffected siblings of probands 1 and 2 (one each) were unavailable for evaluation. Genetic findings and the most significant clinical features of these patients are depicted in Table 1, along with similar data from all previous case reports on children with biallelic *NEUROG3* mutations for comparison [6, 8–11]. Early-onset severe malabsorptive diarrhoea was a common finding in the 11 patients, with enteric anendocrinosis demonstrated in all cases where this condition was investigated on intestinal biopsies. Absence of enteroendocrine cells was not specifically investigated in any of the four patients included in this study, but it has previously been reported in patients with the same *NEUROG3* mutations [6, 8]. Typically, the intestinal failure requires long-term parenteral nutrition initially, but oral feedings are increasingly tolerated and parenteral nutrition can eventually be discontinued. Low fecal elastase or trypsin levels have been reported in some but not all cases, suggesting that pancreatic exocrine insufficiency represents a functional defect secondary to a lack of cholecystokinin and secretin, the enteric hormones that stimulate pancreatic exocrine secretion [10].

**Table 1:** Clinical and molecular findings in patients with NEUROG3 deficiency

	Proband 1	Proband 2	Proband 3	Sibling of proband 3	Ref. [6]	Ref. [6]	Ref. [6]	Ref. [8]	Ref. [9]	Ref. [10]	Ref. [11]
Sex	Female	Male	Female	Male	Male	Male	Male	Female	Female	Female	Female
Age at report	24 years	17 years	18 years	23 years	Deceased at 2 years 11 months (sepsis following orthotopic liver-intestinal transplant at 2 years)	8 years	9 years	7 years	Deceased at 10 months (chronic cholestatic liver disease secondary to parenteral nutrition)	20 months	3 months
Consanguinity	No	No	Yes	Yes	Yes	Not reported	Not known	No	Denied but from same region in Ecuador	Yes	Yes
Mutations in *NEUROG3* gene	c.404T > C/c.404T > C	c.404T > C/c.404T > C	c.319C > A/c.319C > A	c.319C > A/c.319C > A	c.319C > A/c.319C > A	c.278G > T/c.278G > T	c.278G > T/c.278G > T	c.82G > T/c.404T > C	c.367G > T/c.367G > T	c.510dupG/c.510dupG	Homozygous mutation, not reported
Predicted change on NEUROG3 protein	L135P/L135P	L135P/L135P	R107S/R107S	R107S/R107S	R107S/R107S	R93L/R93L	R93L/R93L	E28X/L135P	E123X/E123X	S171fsX68/S171fsX68	Not reported
Birthweight (g)	2,250	2,250	2,575	3,100	2,530	2,720	2,334	1,910	1,960	3,040	Not reported
Congenital malabsorptive diarrhoea	Yes	Yes	Yes	Yes	Yes	Yes	Yes	Yes	Yes	Yes	Yes
Enteric anendocrinosis	Unknown	Unknown	Unknown	Unknown	Yes	Yes	Yes	Yes	Yes	Yes	Yes
Pancreatic exocrine insufficiency	Yes	Yes	No	No	No	No	No	No	Yes	Yes	Not reported
Pancreatic imaging	Hypoplastic pancreas	Normal	No	No	Not reported	Not reported	Not reported	Normal	Normal	Not reported	Not reported
Diabetes mellitus	Yes	Yes	Yes	No	No	Yes	Yes	Yes	Yes	No	Not reported
Age at presentation	3 weeks	13 years	12 years	–	–	8 years	8 years	3 weeks	5 months	–	–
Subtype	Transient neonatal diabetes, relapsed at 6 years	Permanent diabetes	Permanent diabetes	–	–	Permanent diabetes	Permanent diabetes	Permanent neonatal diabetes	Permanent neonatal diabetes	–	–
Serum C-peptide (nmol/l)	0.04 (postprandial)	0.4 (postprandial)	0.4 (fasting)	0.5 (fasting)	Not reported	Not reported	Not reported	0.5 (postprandial)	0.6 (feeding status not reported)	Not reported	Not reported

No evident genotype–phenotype correlation exists between *NEUROG3* mutations and pancreatic endocrine function. NEUROG3 deficiency can lead to a number of different phenotypes of diabetes, including permanent neonatal diabetes, relapsing transient neonatal diabetes and childhood-onset permanent diabetes. In either case, insulin production is partially spared, as indicated by detectable, although relatively low, C-peptide levels. Furthermore, none of the diabetic patients has ever presented with severe hyperglycaemia or ketosis, not even during acute intercurrent illness, which also supports the persistence of some residual endogenous insulin production. This relative insulin deficiency might be due in part to a lack of incretin hormones (glucagon-like peptide 1, glucose-dependent insulinotropic peptide) from the gut, a hypothesis that has not been tested in NEUROG3-deficient patients so far. However, diabetes does not universally present during childhood or young adulthood, as evidenced by the non-diabetic 23-year-old male patient, although it might develop later on. In addition, the same genotype can cause different diabetic phenotypes both between and within kindreds. Overall, the data available suggest that either genetic or non-genetic modifiers are likely to play a role in the penetrance and expressivity of diabetes in *NEUROG3* biallelic mutation carriers.

The presence of some residual endogenous insulin secretion in patients with NEUROG3 deficiency suggests that, in contrast to mice [3], a redundant NEUROG3-independent pancreatic endocrine developmental pathway might exist in humans so that NEUROG3 is not absolutely required for differentiation of pancreatic progenitors into islet cell precursors. In this sense, it has recently been reported that ASCL1B, another bHLH transcriptor factor, initiates the endocrine cell differentiation programme instead of NEUROG3 in zebrafish [12]. Further research in this area will help clarify the exact role of NEUROG3 in human pancreatic development.

In summary, NEUROG3 deficiency produces a rare clinical syndrome characterised by severe malabsorptive diarrhoea from early life and mild diabetes with a variable age of onset. This finding suggests that NEUROG3 is important but not essential for beta cell development and function in humans.

## Abbreviations

bHLH: Basic helix-loop-helix
NEUROG3: Neurogenin 3
